# Supplementary figures and images for: Visual detection is locked to the internal dynamics of cortico-motor control
Source: PLoS Biol. 2020 Oct 20;18(10):e3000898. doi: 10.1371/journal.pbio.3000898 (PMC7598921; doi:10.1371/journal.pbio.3000898)

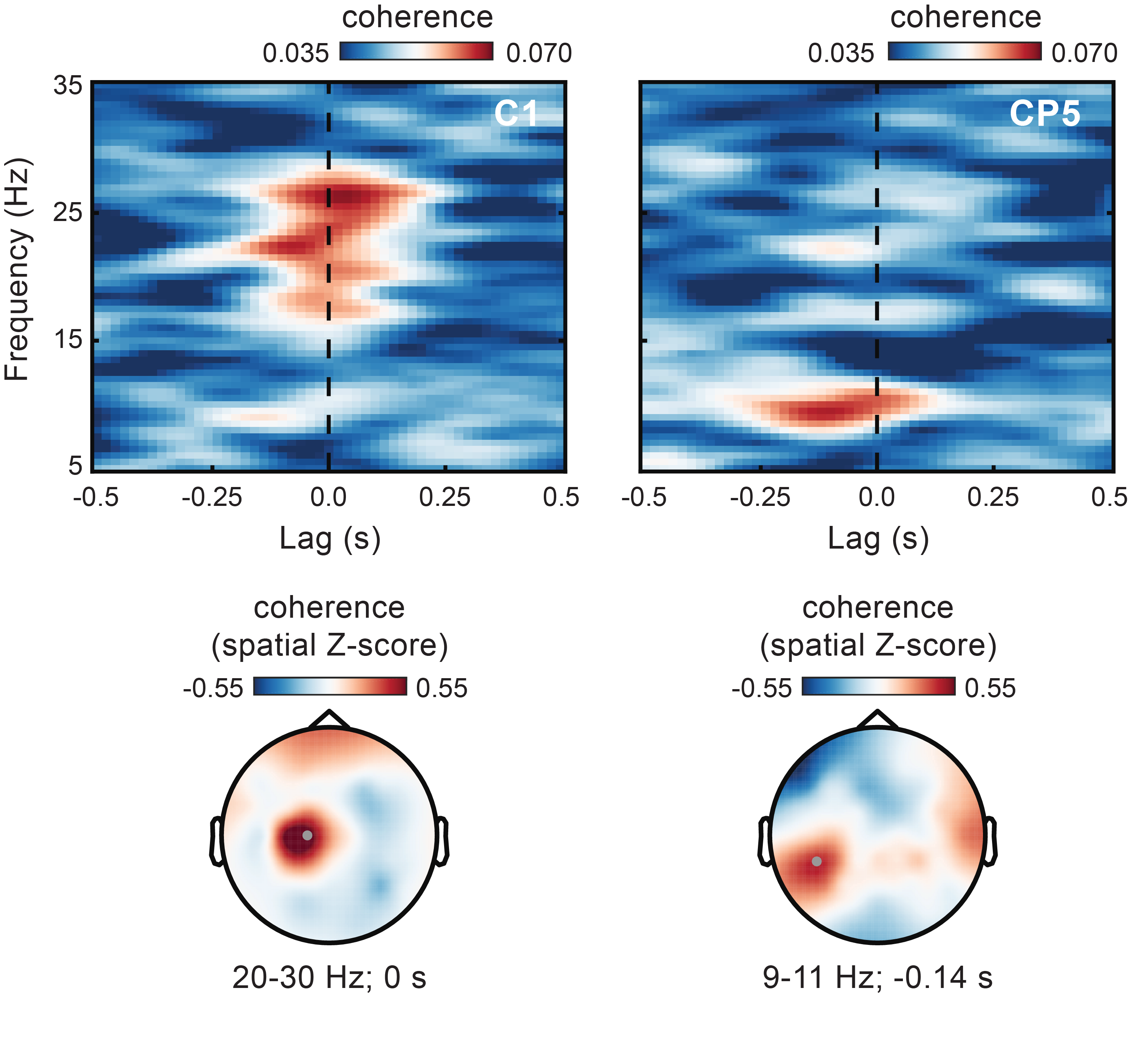

Supplement: S1 Fig — Lag- and frequency-resolved coherence between EEG and EMG activity recorded from a wrist extensor muscle (ECRL). Lagged cortico-EMG coherence is calculated on 0.6-second data windows in the same way as shown in Fig 4A for cortico-force coherence. The raw EMG has been high-pass-filtered at 10 Hz and rectified before computing coherence. Cortico-EMG coherence shows similar properties as those observed for cortico-force coherence. Coherence in the beta-band (20–30 Hz) is symmetrically distributed around zero lag and is maximal over contralateral central electrodes (left topography; C1 marked in gray). Coherence in the alpha-band (9–11 Hz) is biased towards negative lags and distributed over contralateral centroparietal electrodes (right topography; CP5 marked in gray). Notably, alpha-band cortico-EMG coherence peaks at a slightly shorter lag (approximately −0.14 s) compared with its cortico-force counterpart (approximately −0.2 s), which is compatible with the fact that muscular activity anticipates in time its mechanical outcome (i.e., force). ECRL, extensor carpi radialis longus; EEG, electroencephalography; EMG, electromyography. (TIF) [file pbio.3000898.s001.tif]

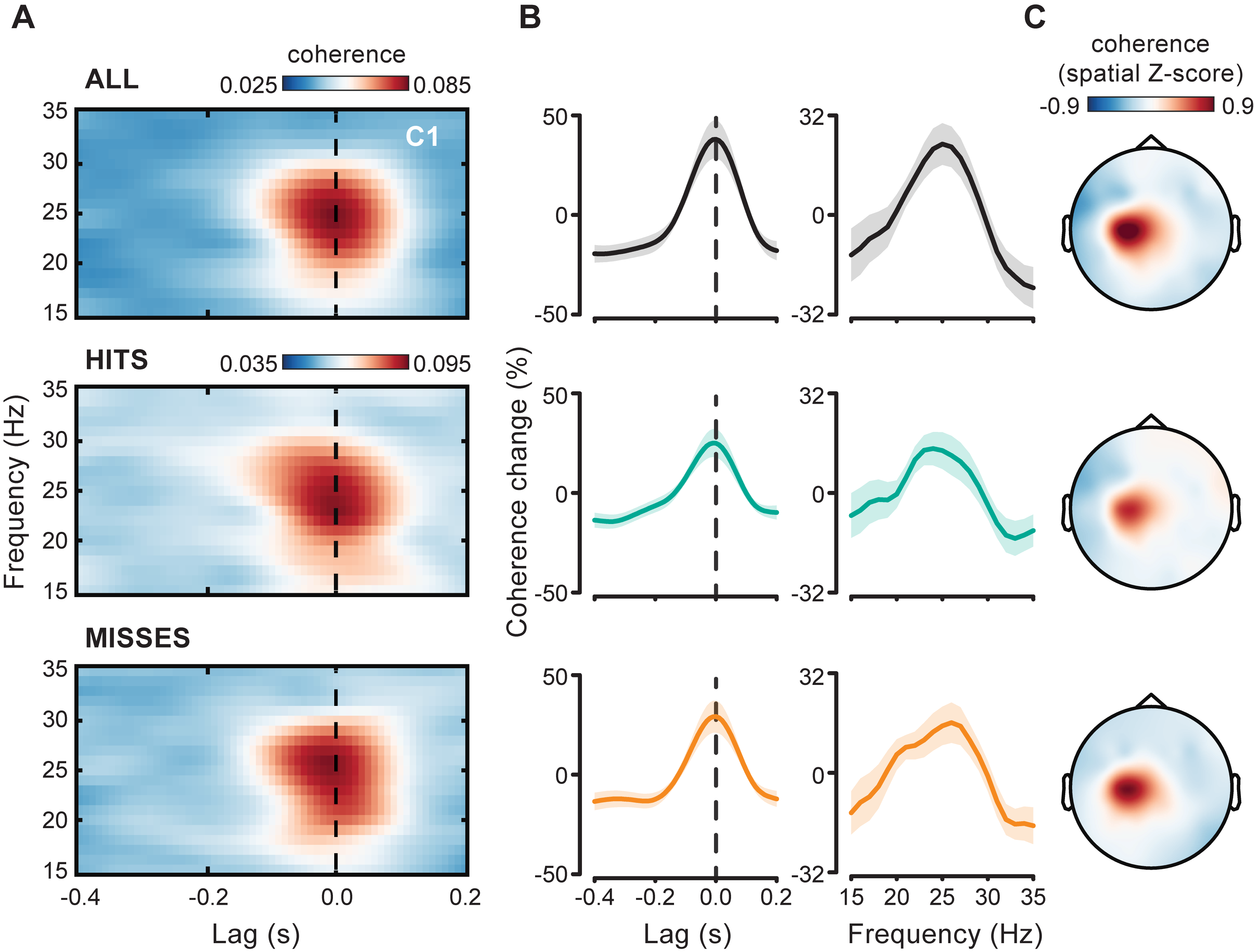

Supplement: S2 Fig — (A) Lag-frequency coherence representation as in Fig 4C but computed on frequencies between 15 and 35 Hz and evaluated at electrode C1 (where coherence in the beta range is maximal). (B) Lag (left) and spectral (right) tuning of beta cortico-force coherence expressed as the relative percentage change in coherence averaged over frequencies between 20 and 30 Hz and lags between −0.08 and +0.08 seconds (i.e., lag of max. beta coherence on all trials [0 seconds] ±1 SD across subjects), respectively. (C) Topographies show coherence at frequency 25 Hz and lag zero for all trials (top), hits (middle), and misses (bottom). (TIF) [file pbio.3000898.s002.tif]

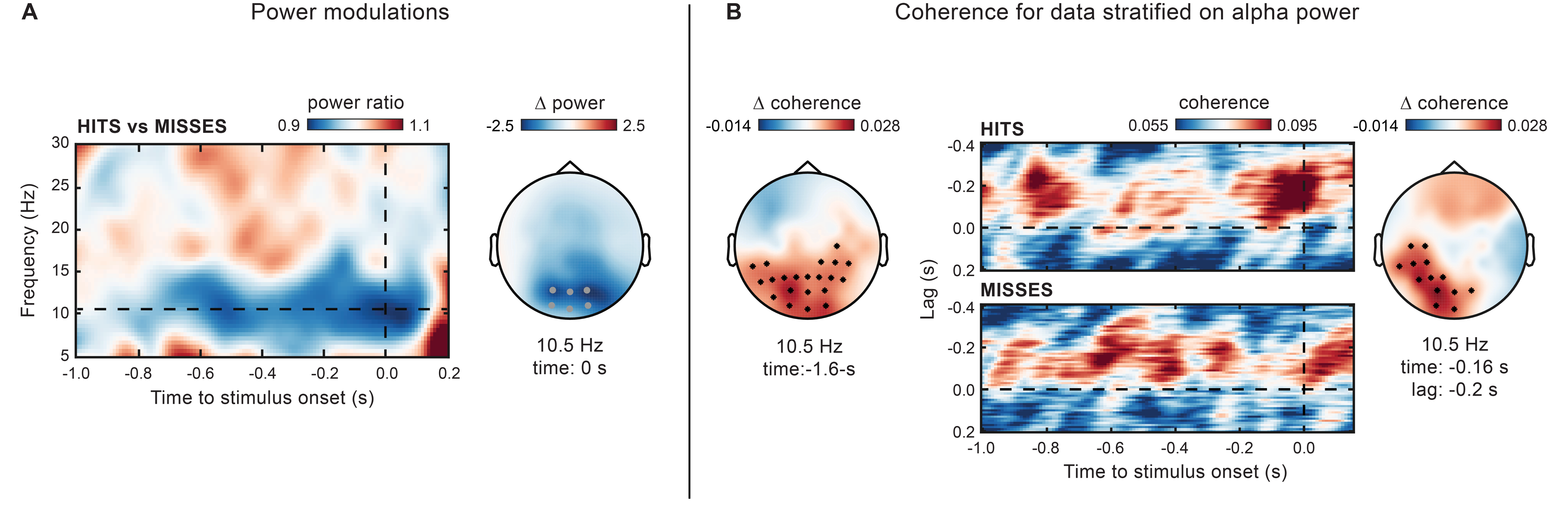

Supplement: S3 Fig — (A) Time-frequency plot of power modulations (hits/misses) averaged over selected occipital EEG electrodes (marked in gray in the topographic map). The topography shows the hits-misses difference in power calculated at stimulus onset (0 s) and at frequency 10.5 Hz. (B) Nonlagged (left; same as in Fig 3) as well as lagged and time-resolved (right, same as in Fig 6) coherence analysis performed on data stratified on occipital alpha power. Specifically, for the stratification we used the power at frequency 10.5 Hz in the relevant time window (left: 1.6-second prestimulus; right: 0.3-second windows from −1 to 0 seconds), averaged over selected occipital channels where the hits-misses difference is maximal (Oz, O1, O2, POz, PO3, PO4; highlighted in gray in A). The power distributions were compiled for hits and misses and then binned in 10 equally spaced bins. The number of trials in each bin for hits and misses was then equated by a random subsampling procedure which aims at matching as much as possible the parameter averages (as implemented in Fieldtrip, function: ft_stratify, method: ‘histogram’, ‘equalbinavg’). This procedure led on average to the removal of 73.05 ± 5 and 76.55 ± 4.4 trials per subject (mean ± SEM) for the first and second analysis, respectively. The difference in coherence between hits and misses after data stratification has then been evaluated by means of a one-tailed cluster-based permutation test at frequency 10.5 Hz and, for the second analysis, at time point −0.16 seconds and lag −0.2 seconds. For both analyses, we obtain significant results: black asterisks mark the electrodes that survived cluster-based permutation test for the hits-misses contrast. EEG, electroencephalography. (TIF) [file pbio.3000898.s003.tif]

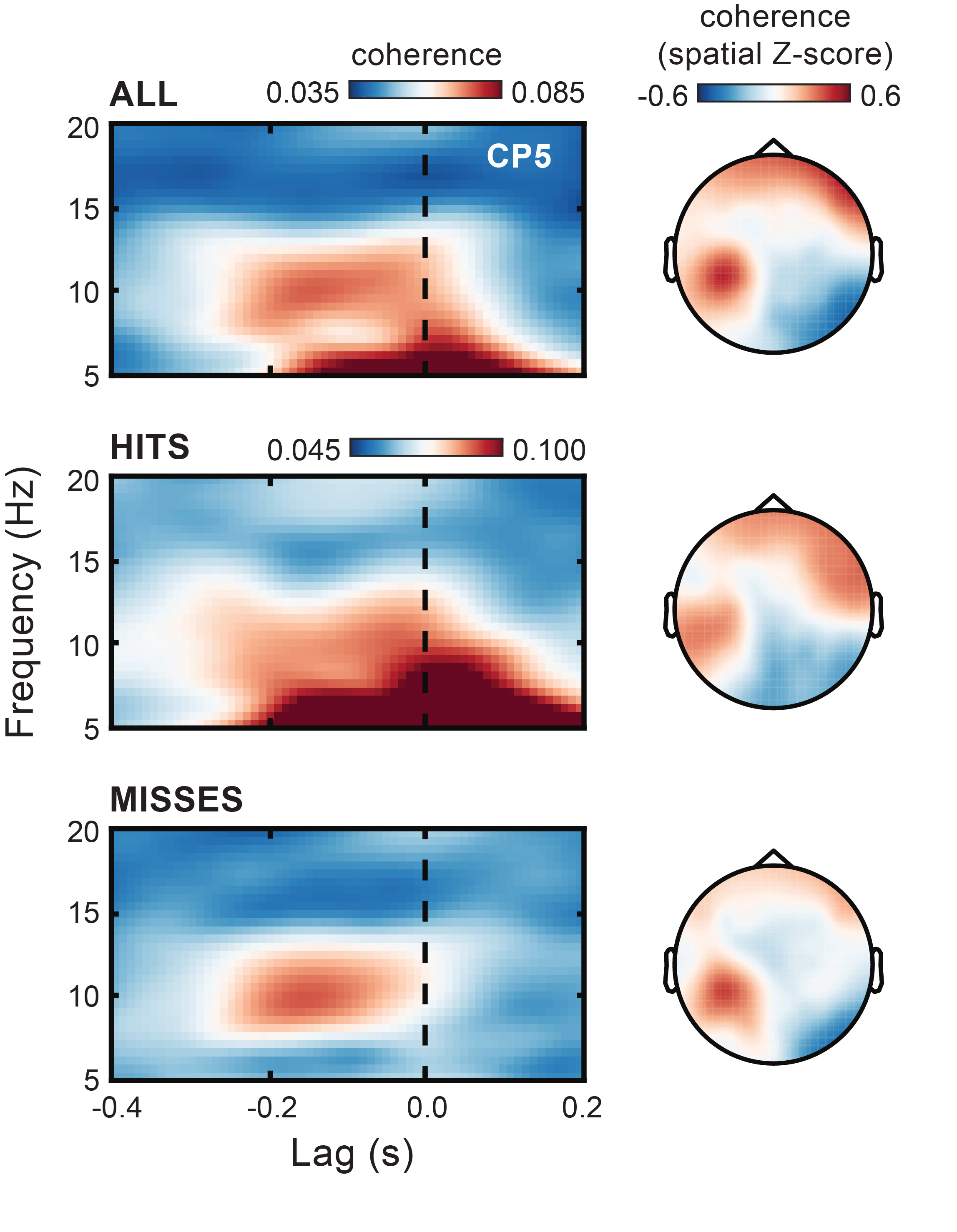

Supplement: S4 Fig — Lag-frequency coherence representation as in Fig 4C but averaged over the poststimulus period (0–0.5 seconds) for all trials as well as separately for hits- and misses-trials. Topographies show corresponding poststimulus coherence (spatially z-scored before averaging across subjects) at 10.5 Hz and lag −0.2 seconds for all trial categories. (TIF) [file pbio.3000898.s004.tif]
