# Supplementary material for: Visual detection is locked to the internal dynamics of cortico-motor control
Source: PLoS Biol. 2020 Oct 20;18(10):e3000898. doi: 10.1371/journal.pbio.3000898 (PMC7598921; doi:10.1371/journal.pbio.3000898)
Supplement: Attachment — Submitted filename: ResponseToReviewers.docx [file pbio.3000898.s005.docx]

Reviewer #1:

I understand this is a first revision. The manuscript is well written and interesting. The authors set out to test the hypothesis that visual information sampling is structured by the internal dynamics of cortico-motor control. They found that alpha band cortico-muscular coherence tended to be elevated just before successful detection of unrelated near-threshold visual stimuli. The authors conclude that "these findings demonstrate an automatic gating of visual inputs by the ongoing motor control processes, providing evidence of an internal and alpha-cycling visuomotor loop."

My main issue with this manuscript lies in the interpretation that assumes, without question, a mechanistic role of alpha band cortico-(force)muscular coherence in gating visual inputs. However, the evidence is correlational and the results could equally be explained by a visual attention related process that is associated with alpha oscillatory activity that then overflows to the motor system. With this perspective there need not be any causal 'coordination' of visual processing by the motor patterning.

We agree with the reviewer that the evidence we provide is “correlational” and not “causal”. In fact, interventional methods (e.g. perturbing motor activity with TMS and observing its effects on visual activity/perception) would be required for concluding that the motor system causes changes in sensory activity/perception. We therefore modified several parts of the text (including the title) to avoid any misleading interpretation with respect to causality. We now claim that our findings provide evidence that visual analysis/perception is *coupled* or *locked* *to* (rather than driven/structured by) the internal motor dynamics.

The reviewer suggests that our findings could be explained by a “visual attention-related process that is associated with alpha oscillatory activity that then overflows to the motor system”. For several reasons, we believe that such an attentional account is unlikely (see point-by-point responses below). Most importantly, we have performed additional analyses that clearly rule out the potential confound due to alpha power differences between hits and misses in the pre-stimulus window. These new results firmly exclude attentional effects associated with alpha power modulations as an alternative explanation of our findings.

On pages 17-19, lines 447-500, we now provide a thorough discussion of the possible mechanistic interpretations of our findings.

The arguments brought to bear against this interpretation are weak:

1."Trial-by-trial fluctuations in arousal and task engagement are expected to yield a positive correlation between perceptual and motor performance (e.g. higher arousal associated with performance improvements in both tasks); within-trial divided attention would instead produce a negative correlation (e.g. attention diverted to the motor task associated with better motor and worse perceptual performance). In contrast, force level and performance in the motor task do not show any type of co-variation with visual performance, suggesting that spontaneous fluctuations in attention/arousal had no major impact on behaviour". But what if the arousal and divided attention are intermixed over time? Then there may well be no net correlation.

In Fig 1C (left) and 1D, we show that perceptual performance (as quantified by hits versus misses) is not associated with any of the possible motor performance variables we could think of.

As suggested by the reviewer, opposite attentional modulations due to arousal and divided attention could still be present but intermixed over time, and therefore cancelling each other and yielding non-systematic effects (no net correlation between visual and motor performance). While we acknowledge this possibility, we don’t think this is likely to be a potential confounding factor for our main results. In fact, if this type of cancellation-by-intermixing would occur, then we would expect it to cancel also the observed association between cortico-force coherence and perception.

Most importantly, we performed new analyses that strongly question a possible attention-based account of our results (see replies to point 2 and 4).

2.Alpha cortico-force coupling is "temporally selective" and this is against attentional processes. This could equally be the product of temporal expectancy and hence an attentional process.

We thank the reviewer for pointing out this aspect that we didn’t sufficiently underline in the previous version of the manuscript.

We now better clarify that temporal expectancy cannot play any role in our study because the visual stimulus was temporally unpredictable due to a 3-s jitter in its appearance (page 18, lines 460-62). Specifically, the presentation time was randomly drawn from a uniform distribution ranging from 1.6 to 4.6 s after the start of the trial; see page 23, lines 577-80. Indeed, such a large temporal jitter was set to prevent participants from effectively preparing in time for the stimulus appearance, thus minimizing potential anticipatory modulations of neural activity.

However, randomly drawing presentation times from a uniform distribution does not completely rule out temporal expectancy effects. In fact, the hazard rate of a uniform distribution increases monotonically with time. To exclude hazard-rate-dependent temporal expectancy effects, we analyzed visual performance as a function of stimulus presentation time (shown below). In a first analysis we used a standard approach based on data binning (10 bins, bin size = 0.3 s) and observed no significant main effect of stimulus presentation time on detection performance (F_9,171_ = 1.401, p = 0.191; one-way ANOVA for repeated measures with stimulus presentation time as within-subject factor). To ascertain that the results did not depend on the (arbitrary) choice of bin size, we also run a second analysis based on logistic regression (i.e., a GLM analysis with a logit link function and a binomial distribution). For each subject we fitted a logistic regression model using as predictor the stimulus presentation time and as dependent variable the visual performance (1-0, hits-misses). We then performed a group-level (random effects) analysis by testing against zero the participant-specific regression coefficients. This analysis confirms the lack of a statistical dependence between visual performance and stimulus presentation time (t_19_ = 1.3607, p = 0.1895; two-tailed one-sample t-test).

In conclusion, we can confidently exclude that temporal attention played a significant role in our study and, most importantly, that it is a confound for the difference in coherence between hits and misses.

Across-subjects average of hits (%) as a function of stimulus presentation time. Gray shaded area represents ± 1 SE.

3."The fast-changing dynamics rules out that the present effect is related to slow fluctuations in arousal or task engagement." Ie cortico-force coherence is temporally confined to a short epoch immediately preceding stimulus onset. This could just be a SNR issue (detection of the tip of the iceberg).

We agree with the reviewer and accordingly removed this statement from the text to avoid invalid conclusions based on the temporal selectivity of the observed effects.

4."Most importantly, the sign of the power modulation (pow hits< pow misses) excludes it as a potential confounding factor for the coherence modulation reported herein (coh hits> coh misses)." This is exactly when coherence can be confounded by power changes. Essentially, if the power of cortical processes that are not coupled to force goes down then relative cortico-force coupling will go up, even though the absolute functional connectivity may be unchanged. Coherence is a relative measure of coupling.

We thank the reviewer as this is a critical point that we didn’t adequately address in the previous version of the manuscript.

Indeed, there are two ways through which power differences (although not statistically significant) between the contrasted conditions (hits and misses) could possibly lead to spurious differences in coherence. In the previous version of the manuscript, we only considered one of them, i.e. that coherence could be boosted in the higher-power condition because of the associated higher signal-to-noise ratio and therefore better phase estimation. This potential confound was ruled out by the fact that the power (misses>hits) and coherence (hits>misses) modulations observed in our study had opposite sign. However, we agree with the reviewer that there is another possibility that leads to the exactly opposite predictions and would therefore be compatible with our results, i.e., high power of alpha activity that is unrelated to the force would obscure the relevant alpha activity, therefore reducing the signal-to-noise ratio of the EEG signals coupled to the force, eventually corrupting phase estimations and producing lower coherence estimates.

To rule out also this possibility (i.e., higher power associated with lower coherence) we used a stratification approach that is aimed at equating as much as possible the distributions of alpha power for hits and misses. We thus repeated both the non-lagged coherence analysis shown in previous Fig 2 (now Fig 3) as well as the lagged (and time-resolved) coherence analysis shown in previous Fig 4 (now Fig 6) on the stratified data. In both cases, the results confirm the original findings (see new S3 Fig), indicating that the difference in coherence between hits and misses is independent from power differences.

More specifically, for the stratification we used the alpha power (at 10.5 Hz) in the relevant time window (first analysis: 1.6-s pre-stimulus; second analysis: 0.3-s windows from -1 to 0 s), averaged over occipital channels (i.e., where the hits-misses difference in alpha power is maximal: Oz, O1, O2, POz, PO3, PO4, highlighted in gray in S3 Fig – previous S2 Fig). The power distributions were compiled for hits and misses and then binned in 10 equally spaced bins. The number of trials in each bin for hits and misses is then equated by a random subsampling procedure which aims at matching as much as possible the parameter averages (as implemented in Fieldtrip, function: ft_stratify, method: ‘histogram’, ‘equalbinavg’). This procedure led on average to the removal of 73.05 ± 5 and 76.55 ± 4.4 trials per subject (MEAN±SE) for the first and second analysis, respectively. The difference in coherence between hits and misses after data stratification has then been evaluated by means of a one-tailed cluster-based permutation test at frequency 10.5 Hz and, for the second analysis, at time point -0.16 s and lag -0.2 s. For both analyses, we obtain significant results (S3 Fig).

These new analyses therefore rule out that alpha power modulations due to attention/arousal could have generated spurious differences in coherence between hits and misses.

5.The phase advance of cortico-force coupling relative to cue presentation by 100-200ms would be more convincing if it were corroborated by other methods of showing "prediction" eg Granger or standard linear regression analysis of phase versus frequency.

Thank you for this suggestion. First, we would like to point out that the EEG signal is phase (time)-advanced relative to the force signal (not relative to the stimulus presentation). As we probably failed in being sufficiently clear on this aspect in the previous version of the manuscript, we have now added a new figure that we hope will help in clarifying how we performed the windowing and the time-shifting of the signals (see new Fig 2).

In accordance with the reviewer’s suggestion, we have performed new analyses aimed at corroborating the driving role of the EEG alpha activity on the force which can be (indirectly) inferred from the lag-selectivity. To this aim we run a frequency-domain Granger causality analysis. Specifically, Granger connectivity was estimated with a non-parametric method: spectral matrix factorization of the cross-spectral density matrix, which was obtained by a Fourier analysis on the entire pre-stimulus interval [-1.6 – 0 s], using the same parameters (multi-tapering, 3-Hz spectral smoothing) as used for the computation of coherence (original Fig 2, now Fig 3). As shown in the new Fig 5, Granger connectivity from the EEG to the force, but not in the opposite direction (from the force to the EEG), shows a clear peak in the alpha range. Moreover, the topography of the EEG-to-force connectivity is very similar to the topography for the coherence coefficient (current Fig 3 & 4). We then tested whether Granger alpha connectivity in both directions is different between hits and misses and found a significant increase in hits compared to misses only for the EEG-to-force connectivity [cluster frequency interval = 9-12 Hz; p = 0.0065]. These results strongly confirm that alpha cortical activity predicts (‘Granger causes’) alpha fluctuations in the force output and this connectivity is stronger before hits compared to misses. We also performed Granger on three non-overlapping 0.5-s epochs, one placed immediately before stimulus onset [from -0.5 to 0 s] and the other two farther away from stimulus onset [from -1 to -0.5 s and from -1.5 to -1 s]. We found that the difference (evaluated at electrode CP5 and frequency 10.5 Hz) between hits and misses is significant only in the epoch closely preceding stimulus presentation [p = 0.0158; see new Fig 7], confirming the temporal selectivity of the coherence modulation.

The manuscript could be crisper. I see little germane in the results section on "Post-stimulus modulations of cortico-force coherence" and the authors fail to entertain what is the most likely explanation for the cortico-force coupling at low frequencies in hits; movement artefact. The Discussion is overlong and uncritical.

Thanks. Following the reviewer’s suggestion, we have substantially modified and shortened several parts of the discussion, to make it more straightforward with respect to the take-home message of the study. On the other side, we have considerably expanded the discussion of the interpretation of our findings, with particular care on the evaluation of the different arguments and available evidence for and against attentional and other alternative accounts (see pages 17-19, lines 447-500).

Concerning the reviewer’s comment on the post-stimulus results:

Previous studies have shown different kinds of stimulus-induced modulations of force and muscle activity during sustained isometric contraction (Novembre et al., 2018, 2019) and dynamic motor tasks (e.g. Wood et al., 2015; Gu et al., 2016). In particular, Novembre and colleagues (2018) reported event-related multi-phasic modulations in the force upon the presentation of salient (high intensity) auditory and somatosensory stimuli. Interestingly, these modulations show trial-by-trial correlations with the concurrent event-related potentials in the EEG activity (vertex potential), suggesting they are unlikely to be mere movement artefacts nor simple reflexive or startle responses (for more details please refer to Novembre et al., 2018). Interestingly, we also observe similar slow event-related modulations in the force for low intensity (near-threshold) visual stimuli and these modulations further scale for the associated perceptual outcome (i.e., they are larger for hits than for misses; see Fig 1C, right). Most recently, Novembre and colleagues (2019) have also reported complex power and phase modulations in the force after stimulus presentation which are strongest in the low frequency range (<10 Hz) and may, at least partly, reflect the event-related modulations observed by the same authors in the time domain (see discussion in Novembre et al., 2019). We believe that the post-stimulus cortico-force coherence observed in the low frequency range for hits may thus likely reflect event-related potentials that co-occur and correlate between the force and the EEG. However, digging into this particular issue would require performing analyses and expanding discussions along a line that largely diverges from the core hypothesis and findings of our study.

In this respect, we feel that an adequate assessment of all the post-stimulus modulations would force us to deviate too much from the focus of the present study. In fact, results on post-stimulus modulations are marginal as they do not provide essential information about the phenomenon of interest, i.e., the relevance of the ongoing dynamics of cortico-motor control for visual perception. This is why these effects were entirely left out from the Discussion section in the previous version of the manuscript. In light of the above considerations and for the sake of clarity and readability of the paper, we decided to restrict the post-stimulus results to the assessment of the lagged alpha component (leaving the other stimulus-evoked modulations aside) and move it to the supplementary materials (see new S4 Fig).

P7, line 153 and Fig 2 Why are the time series of coherence spatially z scored? I can see that this is appropriate for the maps but not the time-series.

If we understand correctly, the reviewer refers to the coherence spectra (not the time-series) shown in previous Fig 2 - now Fig 3 (in fact, no time series are shown in the figure). For visualization purposes (but not for statistics), we initially preferred to maintain correspondence between the quantity shown in the topographic maps (normalized coherence) and the one shown in the coherence spectra (as done also in previous papers: e.g. Schoffelen et al., J Neurosci 2011). Following the reviewer’s suggestion, we have now changed the plots to show the spectra for non-normalized coherence (see Fig 3).

P7, line 169 What is meant by "corrected for frequencies between 5 and 35Hz"?

All frequencies from 5 to 35 Hz were included in the cluster-based permutation test performed on non-lagged coherence (previous Fig 2B, now Fig 3B). Similarly, all time points from -1 to 0 s and all lags from -0.4 to 0 s were included in one single cluster-based permutation test performed on the lagged coherence (previous Fig 4, now Fig 6). This means that we statistically accounted for the multiple comparisons across those three dimensions (for more details on how the cluster-based permutation approach deals with the multiple comparisons problem please see page 29, lines 721-732 and Maris & Oostenveld, 2007).

Frequency selectivity of the modulation of visual perception is claimed but no data shown.

We have now changed previous Fig 2 (now Fig 3) to match the frequency axes in panels A & B and show power (left) and coherence (right) for hits and misses also in the beta range (i.e., up to 35 Hz).

P8 bottom Some comment on the phase lags/leads imposed, if any, by the filters should be included (ideally demonstrated by applying the same processing pathway to surrogate data).

As described in page 28, lines 699-702, we computed the cross-correlation between the EEG (C1, CP5) and the force signals that were previously band pass-filtered (beta: 20-30 Hz; alpha: 8-12 Hz) by applying a two-pass Butterworth filter (2nd order for each single pass). Applying the filter in both the forward and backward direction (two-pass) cancels the phase distortion induced by a single pass (as stated now more explicitly in the main text as well as in the legend of Fig 4).

P8 bottom The correlation between beta-band filtered signals is not symmetrical around zero, although the left shift is clearly not as great as for the alpha activity.

Yes, we now acknowledge this more explicitly (page 9, lines 221-23). We also added additional supporting material showing the lag, spectral and spatial selectivity for beta coherence more in detail (see new S2 Fig). Based on this careful evaluation, we can more confidently say that beta band coherence peaks around lag zero.

Was the hand obscured from view to avoid visual feedback?

Yes. As reported in the Methods section, the joystick handle was covered by a black casing with an aperture in the front to fit the hand, thus preventing participants from receiving any visual feedback of their hand (see page 22, lines 555-57).

Fig 1C the right post-stimulus y axis is labelled in mV, whereas this should be force.

Thanks. The isometric joystick measures the applied force via 4 load cells. The recorded output from the joystick is therefore a voltage signal which is acquired as a continuous analog signal by an acquisition board (USB-1608GX, Measurement Computing; sampling rate, 5000 Hz). We agree, however, that the original label (mV) could be misleading, so we have changed it in “Force (a.u.)”.

Fig 1D slope and slope variability cannot be measured in "force (%)"

As described in page 27, lines 666-72, for each subject we expressed the force as % change from the target force prior to submitting it to a linear least-squares fitting and deriving the slope of the best-fitting function. The slope of the best-fitting function is therefore expressed as force % per second. We have now changed the labels of Fig 1D to make this clearer.

Fig 2: Please explain what is meant by spatial z-score.

The applied spatial normalization is computed for each subject and frequency by subtracting the mean coherence across all the electrodes and then dividing by the standard deviation across the electrodes. We now explain how the spatial normalization was applied in the legend of Fig 3 (page 6, lines 151-53).

Fig 3 and 5. Please fix calibration bars across panels.

Wherever possible, we set equal scales across panels. However, sometimes this was only possible at the cost of substantially obscuring critical information. More specifically, in the following cases the choice of a different scale was done on purpose to allow conveying the most relevant information:

1) Fig 3A (now 4A): the scale is different between the left (C1) and right (CP5) plots because coherence in the beta and alpha range have different absolute values. The main aim of Fig 4A is, however, that of illustrating the lag- and frequency-selectivity of alpha and beta coherence not that of comparing them for their absolute strengths. Equating the two scales would reduce the availability of this information, either by saturating and flattening beta coherence or, vice versa, by making alpha coherence less visible.
2) Fig 3C (now 4C): coherence is a metric with a positive bias (it is always positive for finite samples) and this bias scales with sample size (coherence is higher for smaller samples). Because of this sample-size-dependent bias, coherence computed on all trials is lower than that computed on hits and misses (i.e., half trials). We thus used a different scale for all trials compared to that used for hits- and misses-trials.

As regards Fig 5A (new S4 Fig) we have now set equal scales for hits and misses. For the same reasons described in 2), the scale for coherence on all trials is kept different from that for hits and misses.

In Fig 5 as shown it appears that cortico-force coupling is greater about 200ms before cue presentation in misses than in hits which is at odds with the core result in Fig 4. What is the explanation? In Fig 5 the legend states "Lag-frequency coherence representation as in Fig 3C but averaged over the post-stimulus period (0-0.5 s) for all trials as well as separately for hits- and misses-trials." But a post-stimulus period of 0 to 0.2 is only shown. Please clarify.

We assume the reviewer refers to the previous Fig 5A (now S4 Fig of the supplementary materials). We think we have been insufficiently clear, and this has led to a confusion of the time dimension (which is relative to stimulus onset) with the lag dimension (which is the time shift between the EEG and the force signal). In fact, as reported also in the figure legend, coherence has been averaged over the post-stimulus interval (0-0.5 s); in other words, the data have been collapsed over the time dimension, and therefore this dimension is not shown in the plot. What *is* shown in S4 Fig is instead the lag (and frequency) dependency of coherence: time zero indicates a zero lag between the EEG and the force signal, negative lags (from -0.4 to 0 s) indicate that EEG precedes force, and positive lags (from 0 to 0.2 s) indicate that EEG follows force. Therefore, S4 Fig shows for the post-stimulus data exactly what is shown in Fig 3C (now 4C) for the pre-stimulus data: cortico-force alpha coherence is maximal when the EEG signals are backward shifted by ~200-ms relative to the force signals.

We have now added a new figure (Fig 2) that illustrates how the windowing and time-shifting of signals has been performed in the main analyses.

Reviewer #2:

The study by Tomassini et al. investigates an important and timely issue: whether and how perceptual and motor processes interact temporally. While I generally like the study, some of the results presented do not seem to be relevant to the aims. I also feel that there is an alternate explanation for the findings, which the authors fail to discuss. My major concern is related to this issue, and I have several minor concerns as well, detailed below.

Major: 
1) I see no evidence that the alpha band cortico-force coherence really indexes the motor system. In fact, the topography in supplementary figure 2 is more visual. Therefore, an alternative explanation for the results is intermodal attention. For example, it is feasible to think that visual perception would be enhanced by suppressing other sensory cortical regions (auditory + somatosensory), using well documented alpha suppressive mechanisms (e.g. by Saskia Haegens). Additionally, "suppressive alpha" in sensory cortices could be coherent with motor alpha, similarly to oscillations in other frequency bands (e.g. gamma binding across visual regions or theta entrainment between hippocampus/sensory cortex). This would be an important finding, but the authors need to provide evidence for or against this notion and discuss the issue.

Thanks. As the reviewer correctly points out, alpha activity is well-known to be most prominent over parietal and occipital regions. However, beyond the higher-amplitude posterior alpha rhythm, a distinct sensorimotor alpha rhythm has also long been known and most recently better characterized both anatomically and functionally (e.g. Stolk et al., 2019; Brinkman et al., 2016). Indeed, distinct alpha sources are likely to be distributed over the whole scalp and functionally differentiated (e.g. van der Meij et al., 2016; Billig et al., 2019).

Therefore, despite alpha power being largest over occipital sites, a distinct (perhaps of lower amplitude) alpha activity could reflect motor system activity. Indeed, our results clearly point in this direction by showing that a cortical alpha rhythm (topographically localized over centroparietal electrodes contralateral to the hand effector) ‘drives’ corresponding alpha fluctuations in the motor output anticipating them by about 0.2 s (see also new analyses based on Granger causality, Fig 5-7). In our opinion, the identified alpha activity is therefore, by definition, “motor” activity. Yet, to be deemed “motor” such an alpha activity does not need to be anatomically localized or generated in M1: prominent cortico-spinal projections (the most likely anatomical carrier of cortico-motor coherence) are indeed known to originate from several cortical areas beyond M1 and S1 (Lemon, 2008; premotor cortex: Dum, Strick, 1991; parietal cortex: Rathelot, Dum, Strick, 2017). We now extensively discuss this important point (see pages 17-19, lines 447-500).

The reviewer suggests that this alpha activity may actually originate from sensory cortices (auditory/somatosensory) undergoing active “suppression” or inhibition because of their task-irrelevance, therefore facilitating visual perception. Indeed, many studies have documented that this attention-based suppression is accompanied by alpha power enhancement (Van Diepen et al., 2019 for a review). To explain our findings, this inhibitory alpha activity would then need to drive coherent alpha activity in those cortical areas that project to spinal centers, eventually resulting in cortico-force/muscle alpha coherence.

While the functional significance of the above modulation (i.e., inhibitory sensory alpha driving motor cortico-spinal alpha) would remain to be clarified, we acknowledge that, at least in principle, this is a possibility. However, our data do not seem to be consistent with it. In fact, hits should be preceded by stronger suppression of task-irrelevant areas (signifying successful intermodal attention allocation) compared to misses and therefore higher alpha power. Yet, the trend of the power modulation observed in our data is the opposite: alpha power is lower for hits than for misses (as shown in previous Fig S2, now Fig S3).This pattern in our data is more compatible with the hypothesis that lower alpha power in visual areas is associated with higher visual excitability, yielding perceptual benefits (a phenomenon that has also been well characterized: e.g. van Dijk et al., 2008; Romei et al., 2010; Iemi & Busch, 2018). This latter hypothesis is further supported by the occipital topography of the difference in alpha power between hits and misses (S3 Fig).

Given the evidence in our data, we can envision two main scenarios:

1) Suppression of task-irrelevant areas is present to a similar degree for hits and misses, i.e., it does not significantly predict the perceptual performance.
2) Suppression of task-irrelevant areas is present and also different between hits and misses but it is not captured by our EEG recordings.

If the first scenario holds, alpha power enhancement cannot represent an alternative account for our findings. (It is important to note that we are not interested here in demonstrating the presence/absence of this attentional process per se, but only its potential confounding role for our findings)

The second scenario is unlikely as it contrasts with many previous reports of this phenomenon by means of EEG recordings (Worden et al., 2000; Foxe et al., 1998; Kelly et al., 2006; Fu et al., 2001; Rihs et al., 2007; Kerlin et al., 2010). Anyway, to address this possibility, future studies making use of different neuroimaging techniques (e.g. ECoG, MEG) would be needed.

Finally, and most importantly, all confounds due to possible alpha power modulations have been ruled out by new control analyses based on stratification (see reply to reviewer 1, point 4 and S3 Fig).

In the Discussion section, we now address more carefully the possible mechanisms underlying our findings, including a thoroughly discussion of the potential role of attentional factors (see pages 17-19, lines 447-500).

Minor:
2)Some sections of the manuscript (like the last paragraph of the introduction) are not clearly written.

Thanks. We have now substantially rephrased a large part of the introduction including the description of the main hypothesis of the study (pages 3-4, lines 53-69).

3)What is the purpose of the "Lagged cortico-force coherence" section of the results? How does it contribute to addressing the main question?

Lag-dependency is a fundamental property of the internal dynamics of cortico-motor control – i.e., the phenomenon under investigation here. Lag-dependency informs us about the directionality of the corticospinal connection. Following the suggestion of reviewer 1 (point 5), we have further corroborated the directionality inferred (indirectly) from the observed lag-selectivity with additional analyses based on Granger causality. Specifically, we showed that the alpha connectivity only emerged in the EEG-to-force direction and not in the force-to-EEG direction (see new Fig 5,7). This is of particular interest given that corticospinal coherence in the alpha range has been reported only sporadically (Raethjen et al., 2002; McAuley et al., 1997) and its lag-dependence has never been described before.

4)Related to both above concerns, the use of the term "lag" for both oscillatory phases and stimulus timing related effects is confusing.

We are sorry if we haven’t been sufficiently clear on this important point. Indeed, we never intended to confuse “lag” with “time”. We now better clarify this point with a new figure (Fig 2) that illustrates the exact windowing and time-shifting of the signals that has been applied in the main analyses.

5)While the introduction is very informative and concise (except the last paragraph), the discussion is way too long.

Thanks. We have substantially shortened large parts of the discussion and made it more straightforward with respect to the main take-home message of the study. However, in light of the suggestions and remarks of more than one reviewer, we felt it appropriate to considerably expand the discussion of the possible interpretations of our findings (see pages 17-19, lines 447-500).

6)"in the post-stimulus time interval." Should be added to the sentence starting on line 118.

Done.

7)The two traces in Figure 2B should be overlaid.

Done (current Fig 3B).

8)Are there any statistics that could be done to support differences and similarities in topography claims (e.g. lines 159-161)?

Unfortunately not. When it comes to differences/similarities in topography, we must rely on non-statistical arguments.

9)Line 276: "We also checked that detection-related changes in pre-stimulus oscillatory power could not account for the observed changes in coherence." Which electrode locations were included?

All electrodes were included in the cluster-based permutation test to evaluate the difference in pre-stimulus alpha power between hits and misses (this is so in all analyses unless explicitly stated).

We have now performed also additional analyses that exclude possible confounds due to alpha power modulations (see new S3 Fig and reply to reviewer 1, point 4).

Reviewer #3:

The work by Tomassini and colleagues entitled "Cortico-motor control dynamics orchestrates visual sampling" deals with the very topical and exciting question if central motor rhythms and general perceptual processes are linked. The authors describe a cortico-force coherence in the alpha range (with the cortical signal preceding the motor output) which is stronger for detected visual input. Importantly, this visual input and the exerted force are unrelated and the coherence increases before the visual input.

Overall, the work uses state of the art frequency analysis and statistical approaches. It describes two novel findings (-0.2 lag coherence, difference between hits and misses) with potentially very interesting conclusions.

Unfortunately, at this point it is hard to understand if the conclusion of a pre-stimulus process is justifiable.

We thank the reviewer for his/her positive evaluation of our work. Below we provide evidence that our findings do relate to a genuine pre-stimulus process.

Essential:
1) The authors nicely describe how it is important to understand if the increase in coherence could have been induced by the appearance of the target. Unfortunately, despite studying methods and results, it is hard to follow their reasoning. The authors write that only pre-stimulus data segments until -0.16 are included in the stats (line 265 „[we] therefore restricted the statistical evaluation of coherence to the latest (closest to stimulus onset) time point which encompasses uniquely pre-stimulus data segments (i.e., -0.16 s).". In the methods it is described that „lags from -0.4 to 0.2 s using a 3-cycles (i.e., ~0.286 s) sliding window" were used. Where is the -0.16 s coming from? Should it not be -0.286 s? And how is the lag incorporated? It should be clarified how an overlap with stimulus onset can neither be included in the time period used for calculating the coherence nor the lag.

In order to understand the figures (e.g. fig 4) correctly, it is important to explain more explicitly if the time point plotted refers to the starting point of the time period used for the calculation of coherence (3 cycles of 10.5 Hz) or the central point. Additionally, it needs to be explained how the lag is plotted and if the time point in the figure is based on the EEG or force. This is not important for 0 lag but certainly for the other lags. EEG is preceding the force at -0.2 lag (significant effect); so is at time point 0 not the force already 0.2 after stimulus onset?

We regret that we haven’t been sufficiently clear in explaining the data windowing procedure and the relative time shift (lag) between the EEG and force. Indeed, this aspect has generated some confusion also for other reviewers. We have therefore added more details in the text (page 12, lines 303-8) as well as a new figure that graphically illustrates both the windowing and the time-shifting of signals that have been applied in the main analyses (Fig 2).

The analysis the reviewer refers to is based on the computation of coherence between data segments of ~0.3 s (more precisely: 3 cycles at 10.5 Hz). Each ~0.3-s data window is centered at time points from -1 to 0.15 s (relative to stimulus onset) with incremental steps of 10 ms. Thus, when centered at time -0.16 s, the analyzed data window extends from ~ -0.31 s (i.e., -0.16 – [3 cycles at 10.5 Hz divided by 2]) up to ~ -0.01 s (i.e., -0.16 + [3 cycles at 10.5 Hz divided by 2]). This window therefore includes only data points that precede stimulus onset (before and not including time zero).

The above reasoning is valid for both force and EEG at lag zero. The time-shift procedure was applied to the EEG signals, keeping the force signals fixed in time (as a sanity check, see the results for the reverse time-shift in the reply to reviewer 4, point 4). Therefore, the time axis shown in previous Fig 4 (now Fig 6) indicates the central point of the ~0.3-s data windows of the force signal. The position of the EEG data windows changes as a function of the lag: it coincides with that of the force signal at lag zero, it precedes it at negative lags and follows it at positive lags. This means that at time -0.16 s and lag -0.2 s (where our effect is significant; see bar plot in Fig 6), the analyzed EEG data window extends from ~ -0.51 to -0.201 s (i.e., the position of the force data window backward shifted in time by 0.2 s due to the negative lag). In conclusion, this means that hits and misses are significantly different when considering only pre-stimulus data for both the EEG and the force.

We hope we have resolved any doubt about the pre-stimulus nature of the reported effects and that the new figure will significantly help in making this important point clear. In further support of the pre-stimulus nature of the effect see also the new analyses based on Granger causality (Fig 7).

2) The authors write: " [the stronger cortico-force coherence …] cannot be explained by differences in alpha tremor amplitude which is comparable irrespective of the perceptual outcome (Fig 2B, left).

While this is true there might be another possible explanation namely, that a reduction of near-by alpha oscillations facilitates the detection of the coherence. If strong continuous visual alpha (not phase locked to the alpha that is coherent to the force oscillation) is reduced before a hit (as shown in S2 Fig), this could theoretically lead to a better phase estimation of the cortical alpha that contributes to the coherence. Is it possible to do a correlation between occipital alpha power and coherence strength to exclude this possibility? Overall, the paragraph 276-282 deems insufficient to discuss possible alpha power influences.

Thanks for having raised this important point. Unfortunately, because coherence is calculated across trials, correlating occipital alpha power and coherence cannot be performed at the trial level. To work around this problem and to adequately deal with potential confounds due to power modulations, we adopted a stratification approach. As already extensively described in the reply to reviewer 1 (point 4), we stratified the data based on occipital alpha power (averaged over the electrodes highlighted in gray in S3 Fig, left) and repeated the analysis of the non-lagged (previous Fig 2, now Fig 3) and lagged (previous Fig 4, now Fig 6) coherence on the stratified data. Stratification aims at equating as much as possible the distributions of the potential confounding variable (here, occipital alpha power) between the conditions (here, hits and misses) by means of an iterative random subsampling procedure. As reported in the new supplementary S3 Fig, the pattern of results remains essentially unchanged after stratification on occipital alpha power. This indicates that the observed coherence modulation is not explained by power differences between hits and misses, such as the power differences that can be due to attentional factors (for more details please see reply to reviewer 1, point 4 and pages 12-13, lines 323-41).

More general comments:

In general, I think it is a shortcoming that the manuscript does not more strongly incorporate previous findings about visual perceptual alpha such as lateralization and inhibition and that the different topographies are not discussed in detail. While the difference between hits and misses is maximal over the occipital part and (only slightly) lateralized in Figure 2, the maximal difference between hits and misses in Fig 4 is maximal over central parietal areas contralateral to force and visual input. Does the difference between Fig 2B and Fig 4 topography stem from the different time periods of analysis or the inclusion of certain lags only? (But Fig 4 left diagram does not suggest a change in lag over time). The obvious difference of significant electrodes is not discussed.

Thanks. We have now included an extended discussion of the possible role of visual/attentional modulations of alpha activity in accounting for our results as well as a discussion of the different topographies (17-19, lines 447-500).

Despite alpha power is clearly largest over occipital sites, alpha generators are likely to be distributed across the whole brain (e.g. van der Meij et al., 2016; Billig et al., 2019). However, because it is strongest at the sensor level, occipital alpha might partly affect sensor-level effect topographies corresponding to hits-vs-misses. As the reviewer correctly points out, the contribution from more posterior sites to the hits-vs-misses topography is particularly evident for those analyses (coherence and Granger causality) that have been performed over the entire (1.6-s) pre-stimulus window (Fig 3B and new Fig 5). In the other analyses that have been performed with better temporal/lag resolution (lagged coherence – Fig 6; Granger – Fig 7), effect topographies appear much more selective and very similar to the topography of alpha coherence/Granger-causality (Fig 3A, 4E, 5). One possibility is that ‘contamination’ from occipital alpha is more detrimental for the poorly resolved analyses (where data segments with virtually no coherence modulation also contribute to the overall effect estimation). However, the occipital contribution to the hits-vs-misses coherence topography is visible also after accounting for occipital alpha power modulations (see new S3 Fig), making the above hypothesis less likely. As argued in page 19, another possibility is that parietal visuomotor integration areas (such as the intraparietal sulcus - IPS) that are known to contribute to the corticospinal tract (Rathelot, Dum, Strick, 2017) could thus also contribute to alpha cortico-force coherence and represent a (major or additional) determinant of its perceptual relevance. The present findings certainly call for future studies (e.g. making use of interventional techniques such as TMS) to clarify the exact cortical origin of the alpha activity highlighted herein.

It is very unfortunate that the experiment did not decouple the visual and motor components via lateralization, which would have been easy to do by placing the visual target at the opposite side from the hand that had to do the force task. This would have strengthened the interpretation of a motor-visual interaction considerably.

Thanks for this interesting comment.

Testing both spatial positions (right and left visual hemifields) would allow us to provide information also with respect to the spatial selectivity of the current phenomenon. In fact, our data do not allow to establish whether the latter is modulated by the spatial alignment between the effector and the stimulus (either in body-centered or in external-world reference frames). Testing both the dominant and non-dominant hands would also be particularly interesting given their known motor control asymmetries (Sainburg, 2014). We do believe that all these issues are very important to clarify the actual functional significance of the present phenomenon for online visuo-motor control.

However, while our findings would certainly be further specified by these additional manipulations, they do not crucially depend on those manipulations. Specifically, we believe that the visual stimulus position in the right hemifield does not induce a confound, and this is for the following reasons:

1) The phenomenon of interest occurs in the pre-stimulus window. Thus, it cannot be affected by (lateralized) stimulus-evoked or -induced modulations.
2) The stimulus is unpredictable in time (3-s jitter), therefore preventing participants from effectively preparing for the stimulus appearance. This minimizes any anticipatory, and possibly left-lateralized, modulation of neural (alpha) activity.
3) Sustained visual attention to the right hemifield (where the stimulus is expected) could be more effective before hits compared to misses, leading to left-lateralized alpha power modulations (e.g. Thut et al., 2006; Sauseng et al., 2005). However, the alpha power modulation relevant for stimulus detection is not lateralized in our data (S3 Fig; This observation pertains also to the point 2).
4) Most importantly, we have performed new analyses based on a data stratification approach that rule out possible confounds due to pre-stimulus alpha power modulations (see reply to point 2).

As argued also in the replies to reviewer 1 (point 4) and reviewer 2 (point 1), we can confidently exclude that obvious, and possibly left-lateralized, visual or attention-related modulations of alpha activity represent a confound for the observed pre-stimulus difference in alpha cortico-force coherence between hits and misses. We now take particular care in discussing all these possibilities (see page 17-19, lines 447-500).

Could the reduction in force after a hit (but not a miss) as shown in Fig 1c, explain the 0 lag post stimulus coherency effect? I would be nice if this would be discussed. May be even a single trial correlation would be possible?

We agree that the stimulus-related modulations of force that can be observed in the time domain (Fig 1C) may, at least partly, contribute to what is observed in the frequency domain as (post-stimulus) zero-lag cortico-force coherence. In particular, previous studies have reported that similar stimulus-evoked modulations in the force show trial-by-trial correlation with corresponding event-related potentials in the EEG activity (Novembre et al., 2018). Such co-occurring and correlated event-related modulations may thus yield coherence between force and EEG in the low-frequency range (for a discussion of related phenomena see also Novembre et al., 2019). Unfortunately, single-trial correlation analyses cannot be performed to ascertain this possibility because the coherence coefficient is computed across trials.

As already stated in the reply to reviewer 1, we believe that the post-stimulus modulations deserve further investigation; however, we feel that such an investigation goes largely beyond the scope of the current study. Indeed, post-stimulus modulations (with the exception of those pertaining to alpha lagged coherence) are essentially unrelated to the current phenomenon of interest, i.e., the relevance of the ongoing dynamics of cortico-motor control for visual perception. An adequate assessment and discussion of this additional set of results within the present work would force us to diverge too much from the focus of the study, diluting its take-home message and reducing clarity and readability. Therefore, we deemed it more appropriate to limit the post-stimulus analyses uniquely to the assessment of the lagged alpha component (leaving the other stimulus-evoked modulations aside) and move this piece of the results to the supplementary materials (see new S4 Fig).

Post stimulus effects are suggested to be introduced by the stimulus but the significant effect at 0.23 s is far from a classical visual response and ipsilateral to stimulus and hand. This should be discussed.

We agree that the ipsilateral topography of the zero-lag cortico-motor synchronization observed around 250 ms post-stimulus is not consistent with the known visual evoked responses. Intriguingly, in the study cited also in the reply to the above point, Novembre and colleagues (2018) found that force modulations following a stimulus are characterized by multiphasic responses of very similar shape irrespective of the stimulus modality (somatosensory/auditory). Trial-by-trial correlations of stimulus-evoked cortical and force modulations show an initial (around 110-180 ms) topography that is frontal and ipsilateral to the hand exerting the force (Novembre et al., 2018). Such a topography resembles the topography observed here around 250 ms. The longer latency of the present response might possibly be due to the low intensity (near-threshold) and/or the visual modality of the present stimulus compared to the high intensity auditory/somatosensory stimuli used by Novembre and colleagues. As suggested also by the authors, the observed phenomenon might be part of a supramodal mechanism implementing a rapid, possibly defensive, reaction to sudden stimuli (see discussion in Novembre 2018, 2019).

As argued more extensively above and also in the reply to reviewer 1, because of the marginality of these effects with respect to the phenomenon under investigation here, we opted to restrict the analyses of the post-stimulus window to the assessment of the lagged alpha component and move these results to the supplementary materials (see S4 Fig).

May be I missed it but was the trial number equal for hits and misses?

Yes. For each subject, we set the stimulus contrast at threshold level (i.e., detection rate = 50%) prior to the beginning of the experiment. Moreover, performance was monitored throughout the experiment and visual contrast was adjusted to keep performance near threshold level (see pages 24-25, lines 607-14). Thus, detection rate approaches on average 50%, yielding equal trial number for hits and misses (see page 5, lines 98-101).

Reviewer #4:

The manuscript by Tomassini et al. reported an interesting study in which they simultaneously measured EEG, EMG and behavioral performance in human subjects maintaining an isometric force during a visual detection task. They found that alpha oscillation in motor output is coherent with cortical alpha oscillation and cortical oscillation usually leads peripheral oscillation by ~0.2 s. In addition, their data suggest that the strength of this cortico-force coherence is predictive of visual detection performance. These results are novel and exciting. That said, there are several major concerns that I think the authors should address before I feel the work made significant advance in our understanding of the effects of sensorimotor coupling on visual processing.

We thank the reviewer for his/her positive evaluation of our work.

Major:
1.I am curious to see if the alpha cortico-force coherence modulation of visual processing is constrained in the same hemisphere or this effect is global. The manuscript would be significantly strengthened if authors could present visual stimulus in the left visual field as compared to the right left visual field in current experiments. As such, the visual information will be mostly processed in the right occipital cortex while motor processes are in the left hemisphere.

Thanks for the interesting comment. We have discussed this extensively in the reply to reviewer 3 who raised a very similar point; we therefore refer to the above reply for all the details.

In brief:

We do believe that the spatial/effector selectivity and the hemispheric lateralization are both aspects of extreme interest to clarify the functional significance of the present phenomenon for visuo-motor control. However, these issues do not impinge on the general validity and/or relevance of our findings, but rather represent a potential extension that would be worthwhile investigating in future studies. In the reply to reviewer 3, we report in detail the reasons why the visual stimulus position in the right hemifield does not induce a confound for the present findings. These arguments highlight how we can confidently exclude both lateralized stimulus-evoked/induced modulations (because of the pre-stimulus nature of our results) as well as lateralized alpha power modulations due to visual (spatial/temporal) attention (see results after power stratification; S3 Fig and reply to reviewer 1, point 4) as possible confounding factors.

2.Figure 1C, it would be intriguing to determine if the post-stimulus differences in force are stimulus related or decision related. The authors could plot post-stimulus forces for False Alarms and Correct Rejection trials using a random time point between 1.6-4.6 s as a pseudo stimulus time.

We thank the reviewer for the suggestion. A systematic analysis of the nature of the post-stimulus force modulations is interesting but, unfortunately, severely limited by the relatively low number of catch trials in our study (15%, i.e., corresponding on average to 98±1.8 trials per subject). Among these trials, False Alarms were very rare (often <3, max. number = 6) and totally absent in almost half of the participants (9 out of 20), amounting to an average rate of 1.3±1.7%, as reported in page 5, lines 100-101. Therefore, the small available data set does not allow us to conduct the analysis suggested by the reviewer in an informative and meaningful way.

We would also like to point out that, albeit interesting per se, the question of the decisional or sensory nature of the post-stimulus force modulations is (as far as we can envision) largely unrelated to the main findings of the present study.

3.Figure 2B: why is the frequency range 5-20 Hz instead of 5-35 Hz in figure 2A?

For illustrative purposes, we restricted the frequency range to allow zooming-in into the phenomenon of interest (difference in alpha coherence between hits and misses). This was done in several figures (previous Fig 2B, 3C & D and 5A) by setting the frequency axis between 5 and 20 Hz. Following the reviewer’s suggestion, we have now changed the frequency axis of previous Fig 2B (now 3B) to 5-35 Hz.

4.Line 182: to strengthen the argument, the authors should use EEG in [-1.1 -0.5s] as reference signals and systematically shift force signals. This should yield similar results.

As a sanity check, in accordance with the reviewer’s suggestion, we repeated the same analysis shown in previous Fig 3A (now 4A), this time by keeping the EEG signal fixed [data window from -1.1 to -0.5 s] and shifting the force signal by up to 0.5 s (before and after the EEG in steps of 10 ms). As expected, the results confirm that maximal coherence in the alpha range (right panel in the figure below) is observed when the force follows the EEG by about 0.2 s (positive lags in the graph). Coherence in the beta range appears centered at zero lag (left panel in the figure below). Please see also our reply to reviewer 1, point 5 (and new Fig 5,7) for additional analyses based on Granger causality that corroborate the directional coupling from EEG to force.

Lag- and frequency-resolved cortico-force coherence is shown for the same two EEG electrodes as in main Fig 4A (C1 and CP5). Coherence has been calculated between an EEG 0.6-s data window centered at -0.8 s (i.e., extending from -1.1 to -0.5 s) and 0.6-s force data windows that are shifted (relative to the EEG) by a variable amount of time (negative lags: force precedes EEG; positive lags: force follows EEG).

5.Most results used cortico-force coherence (with figure S2 showed cortico-EMG coherence). Would all results reported here hold when using cortico-EMG coherence, or cortico-force coherence is more robust?

Yes, the reviewer is right, results on cortico-EMG coherence are less clear. Cortico-force coherence is expected to be more robust for a series of fundamental reasons which motivated our choice for recording force in the first place. Indeed, by design, the task required to reach and maintain a certain level of force, and not an EMG-based criterion.

First, the postural configuration and biomechanics of isometric wrist abduction is such that no single muscle activity can produce the required movement. Second, the contribution of several extrinsic and intrinsic hand muscles to wrist abduction is not equivalent to a linear sum of all of them. Rather, their spatio-temporal composition is adjusted via spinal circuitries (Bizzi, Mussa-Ivaldi, Giszter, 1991). Crucially, this implies that the same force output can be produced with different amount of EMG activity recorded from the prime mover muscle (Bernstein, 1967). Third, for every muscle, the surface EMG is a relatively noisy proxy of the descending corticospinal command. All things considered, the force output indexes the global descending drive and is therefore the best proxy for the cortical control of movement (especially in multi-muscle actions).

6.More details about the custom-made isometric joystick should be provided. In particular, how much their hand moved when reaching the predetermined force as it depends on the stiffness of the joystick?

The isometric joystick has a rigid, non-moveable, handle so as to allow measurement of isometric force. Therefore, in accord with the isometric nature of the present task, the effector did not undergo any physical displacement or movement during motor performance, irrespective of the absolute contraction force.

Minor:
1.Line 100: why the sum of hit rate (0.47) and miss rate (0.52) is not 1?

The numbers reported were rounded imprecisely. We now report the exact percentages (see page 5, line 99).

2.During visual detection task, it is unclear if subjects were instructed to stare at the fixation point throughout trial.

Participants were required to maintain central fixation throughout the trial. We now specify more explicitly this important aspect in several parts of the text (e.g. see page 4, line 76).

They could stare at the place where visual stimulus would appear. Did authors track the eye position of subjects?

This is related to Major #1.

Participants were explicitly instructed to refrain from looking at the location where the visual stimulus was expected to appear and maintain fixation on the central cross throughout the trial. Eye movements were recorded by means of vertical and horizontal EOGs. Overall, participants complied with the experimental instructions: saccadic eye movements were virtually absent while blinks were more frequent (as expected due to the 5.5-s trial duration). When present, EEG artifacts related to eye movements were removed with ICA. Furthermore, as already described in page 26, lines 654-55, trials containing eye movements (saccades, blinks) in the 0.25-s window before stimulus onset were discarded from the analysis.

3.Line 150: it is unclear how phase coherence was calculated and phase coherence was not presented in any figures.

Phase coherence (or just coherence as often referred to for brevity in the main text) was computed according to a standard definition (see e.g. Bastos & Schoffelen, 2016). The following formula was applied:

$${Coh}_{x,y}\left( \omega\right)= \frac{\left| S_{x,y}(\omega) \right|}{\sqrt{S_{x,x}\left( \omega\right)S_{y,y}\left( \omega\right)}}$$

where $S_{x,y}(\omega)$ represents the cross-spectral density between signals $x,y$ at frequency $\omega$ averaged over trials, and $S_{x,x}\left( \omega\right)$ and $S_{y,y}\left( \omega\right)$ are the power of signals $x$ and $y$ at frequency $\omega$, respectively.

This quantity is shown in most of the figures (e.g. current Fig 3,4,6). The key statistics, including the main pre-stimulus contrast between hits and misses (Fig 6), were also performed on this quantity.

4.Figure 1D was discussed before figure 1C.

In the results section Fig 1C (left panel) is actually discussed before (page 5, lines 103-4) Fig 1D (page 5, line 111). Afterwards, we discuss Fig 1C (right panel) which pertains to the post-stimulus force modulations.

5.Line 224: why did the authors use 9-12 Hz rather than 8-12Hz as in line 207?

In accordance with the reviewer’s remark and for the sake of internal consistency, we have now changed the figure so as to show the lag-tuning profile for data averaged over the 8-12 Hz range (Fig 4D).

6.Lin 227: Again, how was [-0.22 -0.14s] chosen? Does this range account for variances across subjects?

The lag interval was chosen to be centered on the maximal lag as computed for all trials in the alpha range (now set equal to 8-12 Hz, see the above reply) but with an arbitrary width. Following the reviewer’s suggestion, we have now set the lag interval as equal to the maximal lag over all trials ±1SD across subjects (i.e., [-0.36 - 0 s]; see page 10, lines 241-43). The overall pattern of results is virtually the same as previously shown.

Overall, I think this is an interesting project but more work needs to be done to clarify the significance. I hope my comments are interpreted in a constructive and supportive manner.
