# Supplementary material for: Visual detection is locked to the internal dynamics of cortico-motor control
Source: PLoS Biol. 2020 Oct 20;18(10):e3000898. doi: 10.1371/journal.pbio.3000898 (PMC7598921; doi:10.1371/journal.pbio.3000898)
Supplement: Attachment — Submitted filename: ResponseToReviewers.docx [file pbio.3000898.s007.docx]

According to the Editor and reviewer 3’s suggestion, we have modified the title of the paper in “Visual detection is locked to the internal dynamics of cortico-motor control”.

We thank all the reviewers for their care and effort in the evaluation of our work.
